# Early nutrition and white matter microstructure in children born very low birth weight

**DOI:** 10.1093/braincomms/fcab066

**Published:** 2021-04-01

**Authors:** Julie Sato, Marlee M Vandewouw, Nicole Bando, Dawn V Y Ng, Helen M Branson, Deborah L O’Connor, Sharon L Unger, Margot J Taylor

**Affiliations:** 1 Diagnostic Imaging, Hospital for Sick Children, Toronto, ON M5G 1X8, Canada; 2 Psychology, University of Toronto, Toronto, ON, Canada; 3 Neurosciences & Mental Health, Hospital for Sick Children, Toronto, ON, Canada; 4 Autism Research Centre, Bloorview Research Institute, Holland Bloorview Kids Rehabilitation Hospital, Toronto, ON, Canada; 5 Institute of Biomedical Engineering, University of Toronto, Toronto, ON, Canada; 6 Translational Medicine, SickKids Research Institute, Toronto, ON, Canada; 7 Nutritional Sciences, University of Toronto, Toronto, ON, Canada; 8 Medical Imaging, University of Toronto, ON, Canada; 9 Paediatrics, University of Toronto, Toronto, ON, Canada; 10 Paediatrics, Mount Sinai Health, Toronto, ON, Canada; 11 Division of Neonatology, The Hospital for Sick Children, Toronto, ON, Canada

**Keywords:** very low birth weight, preterm, nutrition, diffusion tensor imaging, white matter

## Abstract

Infants born at very low birth weight (<1500 g) are vulnerable to nutritional deficits during their first postnatal month, which are associated with poor neurodevelopmental outcomes. Despite this knowledge, the impact of early postnatal nutrition on white matter microstructure in children born with very low birth weight has not been investigated. In this prospective cohort study, we employed a whole-brain approach to investigate associations between precise estimates of nutrient intake within the first postnatal month with white matter microstructure at 5 years of age. Detailed information about breastmilk, macronutrient and energy intakes during this period were prospectively recorded for all participants. Multi-shell diffusion and T_1_-weighted MRIs were acquired in 41 children (21 males; mean scan age: 5.75 ± 0.22 years; mean birth weight: 1028.6 ± 256.8 g). The diffusion tensor imaging and neurite orientation dispersion and density imaging models were used to obtain maps of fractional anisotropy, radial diffusivity, orientation dispersion and neurite density indices. Tract-based spatial statistics was used to test associations between metrics of white matter microstructure with breastmilk, macronutrient (protein, lipids and carbohydrate) and energy intake. Associations between white matter microstructure and cognitive outcomes were also examined. Compared to children who did not meet enteral feeding recommendations, those who achieved enteral protein, lipid and energy recommendations during the first postnatal month showed improved white matter maturation at 5 years. Among the macronutrients, greater protein intake contributed most to the beneficial effect of nutrition, showing widespread increases in fractional anisotropy and reductions in radial diffusivity. No significant associations were found between white matter metrics with breastmilk or carbohydrate intake. Voxel-wise analyses with cognitive outcomes revealed significant associations between higher fractional anisotropy and neurite density index with higher processing speed scores. Lower radial diffusivity and orientation dispersion index were also associated with improved processing speed. Our findings support the long-term impacts of early nutrition on white matter microstructure, which in turn is related to cognitive outcomes. These results provide strong support for early postnatal nutritional intervention as a promising strategy to improve long-term cognitive outcomes of infants born at very low birth weight.

## Teaser

Sato et al. report the long-term associations between early neonatal nutrient intake and later white matter microstructure in children born very low birth weight. Their findings are important for understanding white matter development and for guiding early postnatal nutritional interventions in these high-risk children.

## Graphical Abstract

**Graphical Abstract:**
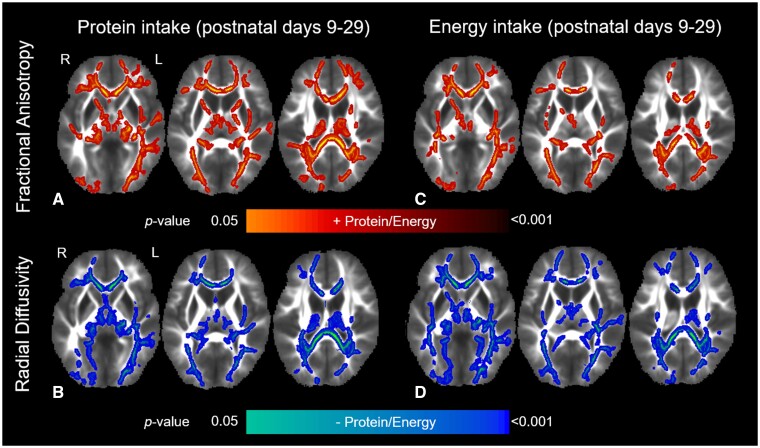

## Introduction

The third trimester, during which very low birth weight (VLBW, <1500 g) or very preterm [<32 weeks gestational age (GA)] infants are born, is a period vulnerable to nutritional deficits in the ex utero environment as placental supply is abruptly discontinued.^1^ These deficits are in turn associated with adverse growth and poor neurodevelopmental outcomes that extend throughout childhood.^2–5^ Thus, an important, modifiable aspect of neonatal care that is critical to improving long-term outcomes is postnatal nutrition in VLBW infants. Emerging evidence suggests that early nutrition, in particular breastmilk and macronutrient intake, is associated with improved neurodevelopment and white matter maturation in preterm infants and adolescents.^5–8^ Despite this knowledge, however, the role of specific nutrients during this critical preterm period on subsequent white matter development at preschool-age has not yet been explored.

Recent studies have shown that higher energy and lipid intake in the first two postnatal weeks were associated with improved white matter maturation and a lower incidence of brain injury at term-equivalent age in very preterm infants.^8^^,^^9^ MRI studies also suggested a dose-dependent relation between in-hospital breastmilk intake in preterm infants and fractional anisotropy (FA) in major white matter tracts at term-equivalent age^6^ and total brain and white matter volumes in adolescence.^5^ The weeks following preterm birth are a period of rapid brain growth and myelination,^10^^,^^11^ which is likely why early nutrient intake in VLBW infants may have long-term impacts on white matter microstructure. Yet there is little literature for understanding the effects of early nutrition on white matter microstructure at preschool-age, a period where cognitive functions are maturing rapidly and amenable to intervention.^12^^,^^13^

Despite advances in neonatal care and increased survival of infants born VLBW, short and long-term cognitive morbidities remain high in this population.^12^^,^^14^^,^^15^ These morbidities are wide-ranging and include cognitive and academic difficulties, as well as impairments in behaviour and social functioning, which typically emerge by school-age.^16–20^ Although several factors have been linked with these poorer neurodevelopmental outcomes, including GA and birth weight, the underlying contributions of white matter microstructure to cognitive outcomes are not well understood. Previous studies have shown that FA increases in early childhood are primarily driven by increases in neurite density index (NDI), suggesting that axon density plays an important role in the maturation of cognitive functions in young children.^21^^,^^22^ Two recent studies reported correlations between both FA and NDI with IQ in school-age children born very preterm.^23^^,^^24^ The authors interpreted these findings to suggest that the association between FA and IQ are related to changes in axon density in children born very preterm.^24^ However, these studies were conducted in older children and did not examine neonatal nutrition as a potential contributing factor to white matter development, and hence also to cognitive sequelae.

In this prospective cohort study, we examined the association between precise estimates of postnatal nutrition, specifically mother’s breastmilk and macronutrient intake, and white matter microstructural parameters in VLBW children at 5 years of age. Diffusion tensor imaging (DTI) and the more recent neurite orientation dispersion and density imaging (NODDI)^25^ model were used to analyse how microstructural properties of white matter were associated with nutrient intake within the first postnatal month. DTI and NODDI are two methods for modelling diffusion-weighted MRI data that have been applied to investigate white matter microstructure in the preterm brain.^23^^,^^24^^,^^26^ We also analysed the associations between DTI and NODDI metrics with cognitive outcomes. Given the known deficits experienced by VLBW children across a range of cognitive domains,^27^ which are strongly associated with the degree of prematurity,^28^ it is critical to understand the underlying contributions from white matter microstructure. We hypothesized that greater nutrient intake would be related to improved measures of white matter microstructure, and that white matter microstructure would in turn would be associated with better cognitive performance.

## Materials and methods

### Participants

Children born VLBW were recruited as part of a 5-year follow-up (NCT02759809) to the randomized clinical trial, Donor Milk for Improved Neurodevelopmental Outcomes (ISRCTN35317141). The study was designed to evaluate the effects of donor milk, in comparison to preterm formula, as a supplement to mother’s breastmilk in infants born VLBW. The feeding protocols and study outcomes have been published.^29–33^ Briefly, 363 infants were recruited from four tertiary neonatal intensive care units in Southern Ontario, Canada, and randomly assigned to receive either donor milk or preterm formula, when mother’s breastmilk was unavailable. Both the feeding intervention and data collection continued after acute care was no longer required and infants were transferred to any one of 17 community neonatal intensive care units in the Greater Toronto and Hamilton areas. The feeding intervention lasted 90 days or until hospital discharge, whichever came first. Study recruitment took place between October 2010 and December 2012. Infants were included if they weighed <1500 g at birth, parents consented within 4 days of birth and if enteral feeding was expected to begin within the first postnatal week. Infants were ineligible if they had severe birth asphyxia or a serious chromosomal or congenital anomaly that could affect neurodevelopment, which were identified before enrolment.

All surviving children (*n* = 316) and their families who took part in the original randomized clinical trial were approached for the 5-year follow-up study. About 158 (50% follow-up rate) participated in the 5-year follow-up between August 2016 and July 2018 at the Hospital for Sick Children (SickKids), Toronto, Canada. This study aimed to recruit 20 VLBW children for each of the three enteral feeding groups during initial hospitalization: (i) donor milk (≥20% of total enteral feeds), (ii) preterm formula (≥20% of total enteral feeds) or (iii) exclusively fed their mother’s breastmilk (i.e. no donor milk or preterm formula supplementation). The study protocol was reviewed and accepted by the SickKids Research Ethics board. All children were screened and approved for MRI compatibility and provided verbal assent while parents gave written informed consent in accordance with the Declaration of Helsinki.

### Clinical and demographic information

Perinatal clinical information was obtained during primary hospitalization in the neonatal intensive care unit, including demographics (birth GA, birth weight, sex and maternal education) and neonatal morbidities [i.e. presence of brain injury, patent ductus arteriosus (diagnosis confirmed by echocardiography or indomethacin treatment), chronic lung disease (oxygen support at 36 weeks corrected age), late-onset sepsis (positive blood or cerebrospinal fluid culture at ≥5 postnatal days) and necrotizing enterocolitis (Modified Bell Staging Criteria ≥ II)]. Clinical radiological review of cranial ultrasounds completed during initial hospitalization at birth defined brain injury as the presence of at least one of the following findings at birth: Echodense intraparenchymal lesions, white matter lesions, periventricular leukomalacia, porencephalic cysts and ventriculomegaly with or without intraventricular haemorrhage. At least two neonatologists and one radiologist assessed the cranial ultrasound scans for the presence of brain injury.

### Macronutrient/energy intake data collection

As part of the feeding intervention, detailed information about macronutrient and energy intakes for all infants were prospectively recorded. These details have been previously published.^29^^,^^32^^,^^34^ Briefly, daily volumes and compositions of the parenteral and enteral nutrition provided were collected for each infant. Mother’s breastmilk intake was calculated as the percentage of total enteral feeds in-hospital. The sum of both parenteral and enteral contributions was used to estimate the daily macronutrient (in grams per kilogram per day) and energy (kilocalories per kilogram per day) intakes. Mean macronutrient and energy intakes were described for the following time intervals: Postnatal Days 1–8 and Days 9–29. These time intervals were selected based on evidence that the first postnatal week (Days 1–8) largely comprises parenteral feeding, as well as significant fluid loss (i.e. diuresis) which can impact weight gain during this period.^35^ Nutrient fortification and full enteral feeding are largely established during Postnatal Days 9–29. Mean intakes were dichotomized based on whether minimum macronutrient/energy for enteral recommendations were met (3.5 g/kg/day for protein; 4.8 g/kg/day for lipids; 11.6 g/kg/day for carbohydrates; 110 kcal/kg/day for energy).^36^ The recommended nutrient intakes were used to facilitate comparisons across studies rather than using cohort-specific cut-offs. Children were excluded if they had <7 days of nutrient intake data available during a given time interval.

### Cognitive assessment

Cognitive performance was assessed using the Wechsler Preschool and Primary Scale of Intelligence-IV (WPPSI-IV),^37^ which is a standardized measure of intelligence that has been validated in children between 2 years and 6 months through to 7 years and 7 months. In addition to the full-scale IQ, other cognitive domains were assessed using the WPPSI-IV, including the Verbal Comprehension Index, Vocabulary Acquisition Index, Visual Spatial Index, Fluid Reasoning Index, Processing Speed Index and Working Memory Index. Composite scores were standardized to a population mean of 100 and a SD of 15, with ‘low average’ scores defined as scores <90.^37^

### MRI data acquisition

MRI scans were acquired on a 3 T MAGNETOM Siemens PrismaFIT with a 20-channel head and neck coil. A T_1_-weighted anatomical image was acquired for each participant with a 3 D magnetization prepared rapid acquisition gradient echo sequence (TR/TE = 1870/3.14 ms, FA = 9°, FOV = 240×256 mm, # slices = 192, resolution = 0.8 mm isotropic). Multi-shell diffusion images were acquired based on the echo planar imaging diffusion pulse sequence [TR/TE = 3800/73 ms, FA = 90°, FOV = 244×244, # slices = 70, resolution = 2.0 mm isotropic, b = 1000/1600/2600s/mm^2^ (30/40/60 directions); 15 interleaved b = 0s/mm^2^ volumes]. A B0 map of the main magnetic field was estimated to correct for distortions in the diffusion data using a double-echo gradient-recalled echo sequence (TR/TE1/TE2 = 600/7.65/5.19 ms, FA = 60°, FOV = 252×252 mm, # slices = 50, resolution = 3.0 mm isotropic). All imaging sequences were collected while children were awake and watching a movie.

### Diffusion processing

Using MRtrix3 and the FMRIB Software Library (FSL), diffusion data were denoized^38^ and corrected for Gibbs-ringing.^39^ Fieldmaps were prepared using FSL’s FUGUE and used in FSL’s eddy tool to correct for motion-induced distortion, echo planar imaging-induced distortions and eddy currents.^40^ The magnitude image was linearly registered (FLIRT) to the diffusion-weighted data and the generated transform was applied to the fieldmap. The eddy toolbox also included an outlier replacement procedure to identify slices with motion-induced signal dropout and replaced them with Gaussian Process predictions.^40^ Diffusion data were corrected for bias field inhomogeneities using the N4 algorithm.^41^ The diffusion tensor was estimated and used to obtain maps of FA and radial diffusivity (RD). The NODDI model was used to obtain maps of orientation dispersion index (ODI) and NDI with the NODDI toolbox for Matlab version 1.0.1.^25^ All three b-values were used to obtain maps of DTI and NODDI metrics. The NODDI model provides additional information about the structural properties of neurites (axons or dendrites) by separating the diffusion signal into three tissue compartments: Intra-cellular, extra-cellular and cerebrospinal fluid.^25^ The intra-cellular compartment measures the diffusion within neurites, from which the ODI and NDI can be calculated. ODI represents the angular variation of neurite orientations, reflecting the organization of axons. NDI estimates the density of axons, particularly myelinated axons.^25^

### Tract-based spatial statistics

A whole-brain approach was taken to analyse diffusion images using FSL’s tract-based spatial statistics (TBSS).^42^ Each child’s FA image was nonlinearly co-registered to every other participant’s FA image, and the most representative FA image was selected as the target image. This target image was then registered to MNI152 standard space and the generated transformations were used to register each participant’s FA data to standard space via the target image. A mean FA template was calculated, skeletonized and thresholded at 0.2 to generate the skeleton mask, which was subsequently applied to each participant’s FA data. The registrations and skeleton mask were also used to obtain skeletonized RD, ODI and NDI data.

### Statistical analyses

#### Participant characteristics

Descriptive statistics were conducted for demographic and clinical variables. Differences between sexes were assessed using an independent samples *t*-test for continuous variables and a chi-square test for categorical variables. Hypothesis tests were two-tailed and *P *<* *0.05 was considered statistically significant.

#### TBSS analyses

FSL’s randomize^43^ was used to perform voxel-wise analyses on DTI and NODDI skeletonized data. In all statistical tests, 5000 permutations and threshold-free cluster enhancement^44^ were used, with significance at *P < *0.05 following corrections for multiple comparisons. In all 41 children, we investigated associations between mother’s breastmilk intake and duration of breastfeeding with DTI and NODDI metrics using a general linear model (GLM), adjusting for both sex and birth weight. In separate models, we tested the association between macronutrient (protein, lipids and carbohydrates) and energy intake with DTI and NODDI metrics, adjusting for both sex and birth GA. Birth weight was not included in these models as there was a significant difference in the mean birth weight between infants who achieved enteral nutrient recommendations during Postnatal Days 9–29 versus those who did not, and thus did not meet the requirements of a covariate.^45^ This is not surprising given VLBW infants are fed based on birth weight, not birth GA. See Supplementary Table 1 for correlation coefficients between confounders. Since <10% of children achieved macronutrient/energy recommendations during Days 1–8, we focussed our analyses on the second time interval of Postnatal Days 9–29. Voxel-wise regression analyses were also performed between DTI and NODDI metrics with cognitive outcomes, adjusting for sex and birth GA.

Exploratory analyses were conducted comparing the type of enteral feeding during initial hospitalization. Children born VLBW were divided into the following three groups based on in-hospital enteral feeding: Those fed exclusively mother’s breastmilk (*n* = 19), those fed mother’s breastmilk supplemented with donor milk (*n* = 13), and those fed mother’s breastmilk supplemented with preterm formula (*n* = 9). The primary exploratory analysis compared the donor milk and the preterm formula group. If no differences were noted between the feeding groups, these two groups were combined and compared with the reference group: Those fed exclusively mother’s breastmilk. To visualize results for significant associations, the John Hopkins University DTI-based tractography atlas^46^ was applied to the FA skeleton to plot values from significant voxels within defined white matter regions. Significant voxels were thickened to help visualize results.

### Data availability

The clinical and demographic data of this study cannot be made available in order to protect the privacy and confidentiality of our participants; we do not have consent from participant families to share their anonymized data, nor do we have permission from the research ethics boards of our participating hospitals. However, the neuroimaging data are available upon reasonable request to the senior author.

## Results

### Participant characteristics

Children were not included into the neuroimaging portion of the study if they were ineligible due to a medical condition (*n* = 3), MRI incompatibility (*n* = 4), their inability to lie still as reported by parents (*n* = 16), or if the anticipated number of participants (i.e. 20) were recruited from their respective enteral feeding group (*n* = 39; see Materials and Methods). Of the 158 VLBW children who attended the 5-year follow-up, 56 consented to a second visit where they underwent an MRI scan and were included in the present analyses. A total of 41 out of 56 children with complete multi-shell scans were included in subsequent analyses after passing inspection for gross motion artefacts. Details on the flow of VLBW participants from the original randomized clinical trial to the present study are summarized in Supplementary Fig. 1. Table 1 summarizes the demographic and clinical characteristics for the total group and sexes separately. There were no significant differences between males and females on any of the demographic or clinical characteristics. Participants had a mean (±SD) age at scan of 5.8 (±0.2) years, birth GA of 28.1 (±2.4) weeks and birth weight of 1028.6 (±256.8) g. Mean (±SD) mother’s breastmilk intake as a percentage of total enteral feeds was 69.7% (±37.1) and ranged from 0.72 (1 infant) to 100% (15 infants). In our cohort, 71% of mothers had an education level of university or above. Of the infants, 12% had a history of brain injury during initial hospitalization, 32% had patent ductus arteriosus, 15% had chronic lung disease and 27% had late-onset sepsis. None of the infants had any incidence of NEC stage II or greater.

**Table 1:** Demographic and clinical characteristics in VLBW children

	Total group (*n* = 41)	Males (*n* = 21)	Females (*n* = 20)	*P*-value^a^
Age at scan	5.8 (0.2)	5.8 (0.2)	5.7 (0.2)	0.15
Gestational age (weeks)	28.1 (2.4)	27.8 (2.5)	28.4 (2.3)	0.43
Birth weight (g)	1028.6 (256.8)	1032.5 (263.7)	1024.5 (256.2)	0.92
Mother’s breastmilk intake (%)^b^	69.7 (37.1)	67.7 (38.6)	71.9 (36.3)	0.73
Duration of breastfeeding (days)	260.6 (187.4)	208.0 (156.9)	315.9 (204.3)	0.06
Maternal education level				
High school	12/41 (29.3%)	8/21 (38.1%)	4/20 (20%)	0.47
University or college	23/41 (56.1%)	8/21 (38.1%)	15/20 (75%)	
Post-graduate training	6/41 (14.6%)	5/21 (23.8%)	1/20 5%)	
Brain injury	5/41 (12.2%)	3/21 (14.3%)	2/20 (10%)	0.68
Patent ductus arteriosus	13/41 (31.7%)	8/21 (38.1%)	5/20 (25%)	0.37
Chronic lung disease	6/41 (14.6%)	3/21 (14.3%)	3/20 (15%)	0.95
Late-onset sepsis	11/41 (26.8%)	6/21 (28.6%)	5/20 (25%)	0.80
Necrotizing enterocolitis (≥2)	0/41 (0%)	0/21 (0%)	0/20 (0%)	–

Categorical variables are presented as frequency (percentage) and continuous variables as mean (SD).

a Comparisons by two-sample *t*-test or chi-square tests.

b Percentage of total enteral feeds.

The mean macronutrient and energy intakes for the VLBW infants during both time intervals (Postnatal Days 1–8 and Postnatal Days 9–29) are shown in Table 2. As described in the methods, only Postnatal Days 9–29 were analysed in since <10% of children achieved macronutrient/energy recommendations during Postnatal Days 1–8. During Postnatal Days 9–29, 28 (70%) children achieved protein and lipid recommendations, 26 (65%) achieved carbohydrate recommendations and 22 (55%) achieved energy recommendations. Mean cognitive scores in our cohort fell within average levels of ability for all measures (Table 3). The proportion of low average scores, defined as IQ scores below 90, was 7 out of 41 (17%) children.

**Table 2:** Mean macronutrient and energy intakes for Postnatal Days 1–8 and 9–29 in VLBW children

	Days 1–8 (*n* = 41)	Days 9–29 (*n* = 40)
Protein (g/kg/day)	2.8 (0.3)	3.7 (0.5)
Lipids (g/kg/day)	2.1 (0.8)	5.0 (1.2)
Carbohydrates (g/kg/day)	9.4 (1.3)	11.9 (1.2)
Energy (kcal/kg/day)	64.9 (10.6)	106.6 (14.8)

Means and SDs are reported.

**Table 3:** Mean cognitive scores in VLBW children

WPPSI-IV indices	VLBW children (*n* = 41)
Full-scale IQ	102.56 (12.97)
Verbal Comprehension Index	101.78 (16.31)
Visual Spatial Index	101.80 (12.64)
Fluid Reasoning Index	101.24 (13.80)
Working Memory Index	103.0 (15.26)
Processing Speed Index	102.34 (11.18)
Vocabulary Acquisition Index	99.33 (15.80)
Low average scores <90, no./total (%) Full-scale IQ	7/41 (17.1%)^a^

Means and SDs are reported.

a Presented as frequency (percentage).

### Associations between early postnatal nutrition with DTI and NODDI metrics

**Figure 1:**
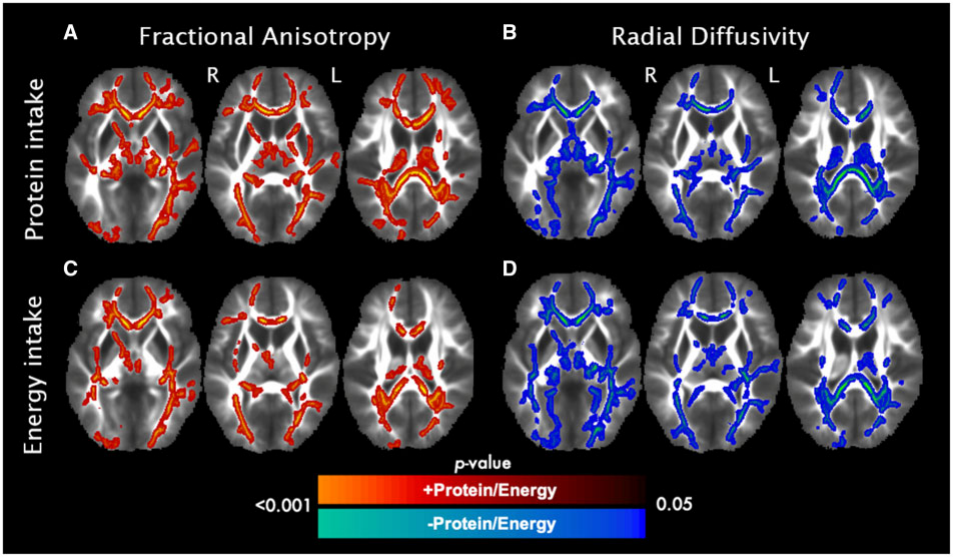
Associations between protein and energy intake with DTI metrics in VLBW children. Red and blue areas represent significant voxels in which higher nutrient intake was positively and negatively associated with DTI metrics, respectively. (**A**) Higher protein intake during Postnatal Days 9–29 was positively associated with FA. (**B**) Higher protein intake was negatively associated with RD. (**C**) Higher energy intake was positively associated with FA. (**D**) Higher energy intake was negatively associated with RD. Significance was held at *P*_corr_<0.05. Colour bars indicate *P*-values.

No significant associations were found between mother’s breastmilk intake with DTI and NODDI metrics in children. The association between duration of breastfeeding with DTI and NODDI metrics was also not significant. Significant associations, however, were present between enteral protein, lipid and energy intakes with DTI metrics (FA and RD), independent of sex and birth GA. The children who met enteral protein recommendations during Postnatal Days 9–29 showed increased FA compared to those who did not (Fig. 1A). Regions with significant voxels included the corpus callosum, corona radiata, anterior limb of the internal capsule and the posterior thalamic radiation (see Table 4 for the full list and number of voxels per subregion that were significantly associated with protein intake). Greater protein intake was associated with lower RD within many overlapping tracts (Fig. 1B andTable 4). Children who met enteral energy recommendations during Postnatal Days 9–29 also showed increased FA (Fig. 1C) and reduced RD (Fig. 1D) compared to those who did not (Table 4). Similar associations were found for lipid intake, albeit less widespread associations with higher FA and lower RD, confined to more posterior tracts such as the posterior thalamic radiation and corpus callosum (Table 4). No significant associations were found between carbohydrate intake with DTI and NODDI metrics.

**Table 4:** TBSS analyses: Associations between DTI metrics with macronutrient and energy intakes in VLBW children

White matter region	Hemi	+FA/+ Protein	−RD/ +Protein	+FA/+ Energy	−RD/+ Energy	+FA/+ Lipids	−RD/ +Lipids
Genu of corpus callosum		1380	1338	923	1103	18	
Body of corpus callosum		1772	1653	1389	1702	1211	1015
Splenium of corpus callosum		1324	1586	1194	1668	1268	1500
Fornix (column and body)		9	13	8	49	1	2
Corticospinal tract	R				1103		
	L				1702		
Cerebral peduncle	R	217	165		94	14	
	L	202	181	64	152	42	
Anterior limb of internal capsule	R	240			4		
	L	223		131	110	114	
Posterior limb of internal capsule	R	264	81	2	88	21	
	L	93	29	51	50	6	
Retrolenticular part of internal capsule	R	94	181	303	454	115	
	L	78	58	150	154	83	96
Anterior corona radiata	R	579	464	313	472		10
	L	748	696	484	617	108	
Superior corona radiata	R	416	437	190	221	352	
	L	293	263	214	352	219	332
Posterior corona radiata	R	479	596	209	311	295	213
	L	454	501	369	518	334	378
Posterior thalamic radiation	R	570	556	478	542	496	317
	L	493	508	508	668	501	454
Sagittal stratum	R	152	179	196	259	166	459
	L	95	8	233	325	128	173
External capsule	R	303	197	322	368	156	11
	L	215	151	93	114	55	121
Cingulum (cingulate gyrus)	R				11	24	
	L	91	93	81	108	101	16
Cingulum (hippocampus)	R				195		101
	L		71		194		
Superior longitudinal fasciculus	R	364	397	185	267	403	241
	L	310	288	48	272	128	201
Superior fronto-occipital fasciculus	R						
	L		2				
Uncinate fasciculus	R	53			44		
	L				27		

This table shows the number of significant voxels per region.

FA = fractional anisotropy; RD = radial diffusivity.

In exploratory analyses, we compared children fed mother’s breastmilk supplemented with donor milk to those supplemented with preterm formula during initial hospitalization. There were no significant differences in DTI or NODDI metrics between the donor milk and preterm formula supplement groups. Thus, these two groups (preterm formula and donor milk) were combined and compared with the reference group: Children who were fed exclusively mother’s breastmilk. However, we also found no significant differences between these groups in any of the DTI or NODDI metrics.

### Associations between DTI and NODDI metrics with cognitive outcomes

**Figure 2:**
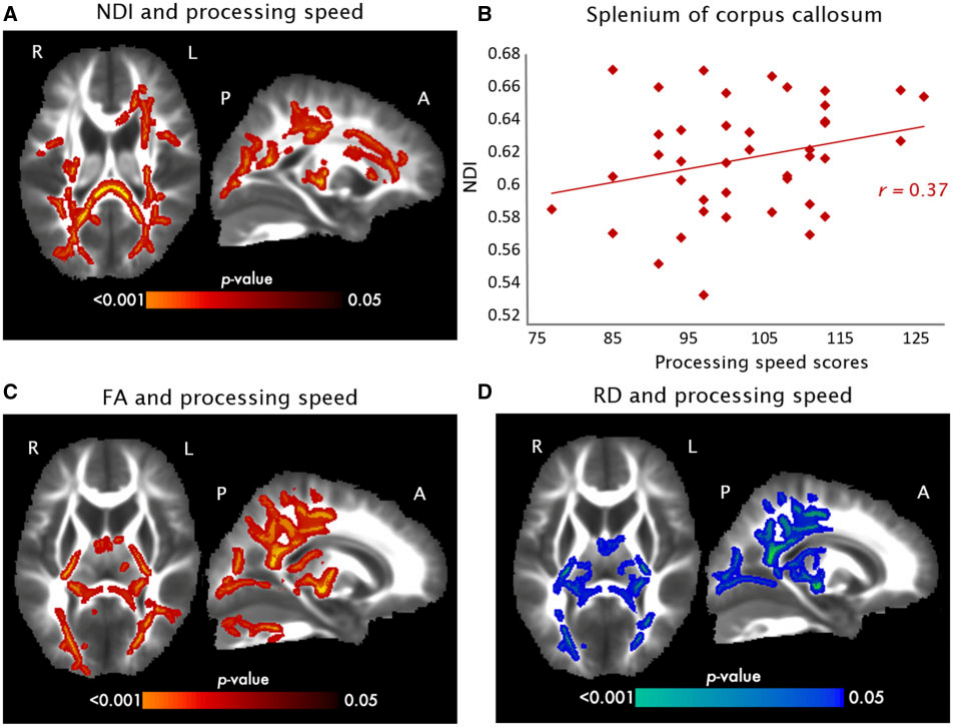
Associations between diffusion and NODDI metrics with processing speed in VLBW children. (**A**) TBSS analyses demonstrated a positive association between NDI and processing speed scores, as indicated by the red areas representing significant voxels. (**B**) The scatter plots show the association between mean NDI in the splenium of corpus callosum and processing speed scores in VLBW children. (**C**) TBSS analyses demonstrated the positive association between FA and processing speed scores. (**D**) TBSS analyses demonstrated the negative association between lower RD and improved processing speed scores, as indicated by the blue areas. Significance was held at *P*_corr_<0.05. Colour bars indicate *P*-values.

Significant associations were found between DTI and NODDI metrics with processing speed scores. Higher NDI (Fig. 2A and B) and FA (Fig. 2C) was associated with higher processing speed in many overlapping regions, including the corpus callosum and the posterior thalamic radiation (Supplementary Table 2). Lower RD was associated with improved processing speed in many of the same tracts where higher FA and NDI were found (Fig. 2D). Lower ODI was also negatively associated with processing speed, however, this association was found in a cluster in the corpus callosum only (Supplementary Fig. 2). No significant associations were found between DTI and NODDI metrics with other cognitive outcomes.

## Discussion

To our knowledge, this is the first study to investigate associations between early postnatal nutrition and white matter microstructure in preschool-age children born VLBW. We leveraged detailed nutritional data collected during neonatal intensive care unit stays following birth to determine the longer-term associations with brain maturation. We found significant associations between early macronutrient intake and white matter microstructure at 5 years of age. Specifically, children who achieved enteral protein, lipid and energy recommendations during Postnatal Days 9–29 showed improved white matter microstructure (increased FA and reduced RD) compared to children who did not meet nutrient recommendations. These effects were independent of sex and birth GA, which are known predictors of neurodevelopmental outcomes in children born VLBW. The DTI and NODDI metrics were, in turn, correlated with cognitive outcomes. Voxel-wise analyses revealed a significant positive association between increased FA and NDI with higher processing speed scores. Lower RD and ODI were also associated with improved processing speed. Importantly, we did not find significant associations between mother’s breastmilk intake or duration of breastfeeding with white matter microstructure.

Our findings demonstrated that among the macronutrients, achieving protein recommendations during Postnatal Days 9–29 contributed to the beneficial effect of nutrition on white matter microstructure at 5 years of age. This was evident by the widespread associations between protein intake with higher FA and lower RD in many overlapping white matter tracts. Proteins are essential for brain development and myelination during the early postnatal preterm period,^47^ a time when the brain is vulnerable to nutritional deficits in VLBW infants. These results significantly extend previous findings showing an association between higher protein intake and greater head circumference growth in infants born very preterm at 2 years of age.^48^ Although not a direct measure of brain development, head circumference is correlated with brain growth at term-equivalent age and is associated with later cognitive and motor outcomes in preterm infants.^49^ In line with these findings, a recent study showed that enteral protein, lipid and caloric intakes during the first postnatal month were positively associated with FA in the posterior limb of the internal capsule.^7^ The authors also found that cumulative protein intake was associated with higher cognitive and motor scores at 2 years corrected age, independent of many risk factors including birth weight, birth GA, sex, white matter injury and illness severity.^7^ Importantly, this study found that days of parenteral (i.e. intravenous) nutrition were associated with poorer white matter maturation and smaller total brain volume. Recently, another study found that higher early parenteral intake of protein within the first postnatal week was associated with an increased odds of cerebral palsy at 7 years of age.^50^ These studies highlight the importance of distinguishing parenteral and enteral sources of nutrition and emphasize the need for further large-scale, randomized trials to determine the optimal dose and timing of enteral protein supplementation in the neonatal preterm period.^51^^,^^52^

We further found that meeting enteral lipid and energy recommendations during Postnatal Days 9–29 was associated with higher FA and lower RD in major posterior white matter tracts such as the posterior thalamic radiation and corpus callosum. These results extend previous findings showing associations between lipid and energy intake with improved white matter maturation and reduced brain injury in preterm infants at term-equivalent age.^7–9^ Our results are the first to extend these findings beyond infancy in children born VLBW, demonstrating the long-term impact of early nutrition on brain development. Lipids play an important role in brain development, serving as the building blocks for membrane formation and myelin synthesis.^53^ This is especially critical in very preterm infants as the oligodendrocytes are particularly vulnerable to damage early in the third trimester when these infants are born.^54^ For instance, brain injury during this preterm period is known to impact the maturation of pre-oligodendrocyte glial cells, which are responsible for the myelination of axons.^55^

Long-chain polyunsaturated fatty acids have also been linked to the beneficial effects of lipids on white matter maturation and neurodevelopment.^56^^,^^57^ Mother’s breastmilk contains precursors of long-chain polyunsaturated fatty acids, which may partly explain the beneficial effect of breastfeeding on neurodevelopment. A Cochrane review, however, found no clear benefit of long-chain polyunsaturated fatty acid supplementation on long-term outcomes in formula-fed preterm infants.^58^ Despite these reports, breastfeeding compared to formula-feeding in preterm infants has been consistently linked with better cognitive performance and increased white matter development in childhood and adolescence.^5^^,^^6^^,^^59^ The lack of a significant relation between mother’s breastmilk intake and white matter microstructure in our study was likely due to the high percentage of breastmilk intake across our VLBW cohort, with half of our sample receiving >90% mother’s breastmilk intake during hospitalization. Earlier studies that found effects, have typically had a greater range of breastfeeding.^5^^,^^6^^,^^60^ In exploratory analyses, significant differences in white matter microstructure were not found between children who received supplementation of donor milk versus preterm formula during initial hospitalization. Previous randomized and observational studies assessing the effect of donor milk compared to preterm formula on neurodevelopment have found similar or slightly lower cognitive scores in those receiving supplemental donor milk.^30^^,^^61^ Our study did not show any significant differences between these supplement feeding groups, although this may be due to the small sample size and thus warrants further investigation. Further, no differences were found between the supplement feeding groups and children who received exclusively mother’s breastmilk, which again may be related to the high intake of mother’s breastmilk in those groups as well during hospitalization.

The associations between white matter microstructure and cognitive outcomes were also very interesting. Alterations in FA and other DTI metrics have been correlated with early cognitive and motor outcomes in very preterm children,^62^^,^^63^ highlighting the importance of understanding which factors contribute to these FA differences. In our cohort of VLBW children, higher FA and NDI were associated with improved processing speed scores. This is consistent with the maturational trends of these distinct metrics, showing general increases with age across white matter regions.^21^^,^^22^ These findings suggest that alterations in axon density may underlie some of the difficulties in cognition observed in VLBW children. In line with these findings, another study also found associations between NDI and cognitive outcomes, specifically IQ and visuomotor skills.^23^ Processing speed is thought to underlie many higher-order cognitive abilities^64^^,^^65^ and has been shown to be impaired in preterm children.^66^^,^^67^ Thus, slower processing speed observed in this population may be related to poorer development of white matter microstructure. Further, the association with FA and RD measures was found posteriorly, which may support a slower posterior-to-anterior gradient of white matter development in children born VLBW. However, given the cross-sectional nature of this work, future studies should replicate these findings in larger studies and longitudinal designs to confirm brain-behaviour associations and developmental delays in VLBW children. In addition, these results suggest a vulnerability of myelination processes, as reflected by associations between processing speed with both RD and NDI, which may help to identify VLBW children at greater risk of later cognitive difficulties.

While the present study has many strengths, including the use of DTI and NODDI metrics to assess the microstructural properties of white matter in children born VLBW, there are some limitations to consider. Firstly, the relatively small sample of 41 VLBW children was due to a combination of factors, including parental decline to participate and ineligibility for neuroimaging. Our sample size was further reduced due to the exclusion of data that did not pass strict inspection for motion artefacts or were incomplete due to the young participants’ inability to finish scanning protocols. While this data loss is a common challenge faced by others working in paediatric neuroimaging, we acknowledge that our sample may not be representative of all VLBW children. For instance, parents who deemed their children unable to lie still during the scan (*n* = 16) did not consent to neuroimaging. Thus, lower functioning VLBW children, who were unable to participate or complete the scan protocol, were not represented in the current sample. Faster scan times and improved scan protocols adapted for paediatric populations will improve the retention and quality of data in the future. Secondly, as is typical in the field, nutrient concentrations of mother’s breastmilk feeds were not directly measured. Weekly macronutrient and energy estimates were instead calculated based on known changes in milk composition over time.^32^ Thirdly, TBSS confines the analysis to the FA skeleton (thought to reflect the centre of major white matter tracts) and is thus not representative of the entire white matter tract, including poor coverage of the cerebellum. While this attempts to exclude voxels with partial volumes and improve alignment between images, we acknowledge this as potential limitation and encourage replication of findings using other analytical methods. Finally, TBSS and NODDI do not account for fibre crossings, and thus fibre-specific analyses could be used in future analyses to complement these results.

Our findings address an important gap in the literature, examining the impact of specific nutritional factors on white matter microstructure measured at preschool-age. We also examined the association between DTI and NODDI metrics with cognitive outcomes. Given the critical role white matter development has on brain function and the many reports of altered white matter in children born VLBW, our study indicates a potential underlying contributor of this impaired white matter maturation. Importantly, our study also highlights the importance of macronutrients such as protein and lipids during this early postnatal period. With the high rates of cognitive difficulties experienced by infants born preterm,^14^^,^^68^ that are not decreasing despite improved neonatal care,^69^ these results provide a target for nutritional interventions in VLBW and very preterm populations, that may help improve long-term outcomes.

## Supplementary material

Supplementary material is available at *Brain Communications* online.

## Supplementary Material

fcab066_Supplementary_Data

## Abbreviations

DTI =: diffusion tensor imaging
FA =: fractional anisotropy
FSL =: FMRIB Software Library
GA =: gestational age
NDI =: neurite density index
NODDI =: neurite orientation dispersion and density imaging
ODI =: orientation dispersion index
RD =: radial diffusivity
TBSS =: tract-based spatial statistics
VLBW =: very low birth weight
WPPSI-IV =: Wechsler Preschool and Primary Scale of Intelligence-IV

## References

[1] Senterre T , Rigo J. Optimizing early nutritional support based on recent recommendations in VLBW infants and postnatal growth restriction. J Pediatr Gastroenterol Nutr. 2011;53(5):536–542. 21701404 10.1097/MPG.0b013e31822a009d

[2] Ehrenkranz RA. Growth in the neonatal intensive care unit influences neurodevelopmental and growth outcomes of extremely low birth weight infants. Pediatrics. 2006;117(4):1253–1261. 16585322 10.1542/peds.2005-1368

[3] Raghuram K , Yang J, Church PT, et al; for the Canadian Neonatal Network. Head growth trajectory and neurodevelopmental outcomes in preterm neonates. Pediatrics. 2017;140(1):e20170216. 28759409 10.1542/peds.2017-0216

[4] Ramel SE , Demerath EW, Gray HL, Younge N, Boys C, Georgieff MK. The relationship of poor linear growth velocity with neonatal illness and two-year neurodevelopment in preterm infants. Neonatology. 2012;102(1):19–24. 22441508 10.1159/000336127

[5] Isaacs EB , Fischl BR, Quinn BT, Chong WK, Gadian DG, Lucas A. Impact of breast milk on intelligence quotient, brain size, and white matter development. Pediatr Res. 2010;67(4):357–362. 20035247 10.1203/PDR.0b013e3181d026da PMC2939272

[6] Blesa M , Sullivan G, Anblagan D, et al Early breast milk exposure modifies brain connectivity in preterm infants. Neuroimage. 2019;184:431–439. 30240903 10.1016/j.neuroimage.2018.09.045

[7] Coviello C , Keunen K, Kersbergen KJ, et al Effects of early nutrition and growth on brain volumes, white matter microstructure, and neurodevelopmental outcome in preterm newborns. Pediatr Res. 2018;83(1):102–110. 28915232 10.1038/pr.2017.227

[8] Schneider J , Fumeaux CJF, Duerden EG, et al Nutrient intake in the first two weeks of life and brain growth in preterm neonates. Pediatrics. 2018;141(3):e20172169. 29440285 10.1542/peds.2017-2169

[9] Beauport L , Schneider J, Faouzi M, et al Impact of early nutritional intake on preterm brain: A magnetic resonance imaging study. J Pediatr. 2017;181:29–36.e1. 27837953 10.1016/j.jpeds.2016.09.073

[10] Kostovic I , Vasung L. Insights from in vitro fetal magnetic resonance imaging of cerebral development. Semin Perinatol. 2009;33(4):220–233. 19631083 10.1053/j.semperi.2009.04.003

[11] Qiu A , Mori S, Miller MI. Diffusion tensor imaging for understanding brain development in early life. Annu Rev Psychol. 2015;66:853–876. 25559117 10.1146/annurev-psych-010814-015340 PMC4474038

[12] Mangin KS , Horwood LJ, Woodward LJ. Cognitive development trajectories of very preterm and typically developing children. Child Dev. 2017;88(1):282–298. 27364183 10.1111/cdev.12585

[13] Karbach J , Kray J. How useful is executive control training? Age differences in near and far transfer of task-switching training. Dev Sci. 2009;12(6):978–990. 19840052 10.1111/j.1467-7687.2009.00846.x

[14] Woodward LJ , Moor S, Hood KM, et al Very preterm children show impairments across multiple neurodevelopmental domains by age 4 years. Arch Dis Child Fetal Neonatal Ed. 2009;94(5):F339–F344. 10.1136/adc.2008.146282 19307223

[15] Burnett AC , Anderson PJ, Lee KJ, et al; for the Victorian Infant Collaborative Study Group. Trends in executive functioning in extremely preterm children across 3 birth eras. Pediatrics. 2018;141(1):e20171958. 29196505 10.1542/peds.2017-1958

[16] Borchers LR , Bruckert L, Travis KE, et al Predicting text reading skills at age 8 years in children born preterm and at term. Early Hum Dev. 2019;130:80–86. 30708270 10.1016/j.earlhumdev.2019.01.012 PMC6402954

[17] Aarnoudse-Moens CSH , Weisglas-Kuperus N, van Goudoever JB, Oosterlaan J. Meta-analysis of neurobehavioral outcomes in very preterm and/or very low birth weight children. Pediatrics. 2009;124(2):717–728. 19651588 10.1542/peds.2008-2816

[18] Aarnoudse-Moens CSH , Oosterlaan J, Duivenvoorden HJ, Van Goudoever JB, Weisglas-Kuperus N. Development of preschool and academic skills in children born very preterm. J Pediatr. 2011;158(1):51–56. 20708749 10.1016/j.jpeds.2010.06.052

[19] Bhutta AT , Cleves MA, Casey PH, Cradock MM, Anand KJS. Cognitive and behavioral outcomes of school-aged children who were born preterm: A meta-analysis. JAMA J Am Med Assoc. 2002;288(6):728–737. 10.1001/jama.288.6.728 12169077

[20] Scott A , Winchester SB, Sullivan MC. Trajectories of problem behaviors from 4 to 23 years in former preterm infants. Int J Behav Dev. 2018;42(2):237–247. 29430071 10.1177/0165025417692899 PMC5805147

[21] Genc S , Malpas CB, Holland SK, Beare R, Silk TJ. Neurite density index is sensitive to age related differences in the developing brain. Neuroimage. 2017;148:373–380. 28087489 10.1016/j.neuroimage.2017.01.023

[22] Mah A , Geeraert B, Lebel C. Detailing neuroanatomical development in late childhood and early adolescence using NODDI. PLoS One. 2017;12(8):e0182340. 28817577 10.1371/journal.pone.0182340 PMC5560526

[23] Young JM , Vandewouw MM, Mossad SI, et al White matter microstructural differences identified using multi-shell diffusion imaging in six-year-old children born very preterm. NeuroImage Clin. 2019;23:101855. 31103872 10.1016/j.nicl.2019.101855 PMC6737393

[24] Kelly CE , Thompson DK, Chen J, et al Axon density and axon orientation dispersion in children born preterm. Hum Brain Mapp. 2016;37(9):3080–3102. 27133221 10.1002/hbm.23227 PMC5524572

[25] Zhang H , Schneider T, Wheeler-Kingshott CA, Alexander DC. NODDI: Practical in vivo neurite orientation dispersion and density imaging of the human brain. Neuroimage. 2012;61(4):1000–1016. 22484410 10.1016/j.neuroimage.2012.03.072

[26] Pecheva D , Kelly C, Kimpton J, et al Recent advances in diffusion neuroimaging: Applications in the developing preterm brain [version 1; referees: 2 approved. F1000Research. 2018;7:1326. 10.12688/f1000research.15073.1 PMC6107996 30210783

[27] Anderson PJ. Neuropsychological outcomes of children born very preterm. Semin Fetal Neonatal Med. 2014;19(2):90–96. 24361279 10.1016/j.siny.2013.11.012

[28] Xiong T , Gonzalez F, Mu DZ. An overview of risk factors for poor neurodevelopmental outcome associated with prematurity. World J Pediatr. 2012;8(4):293–300. 23151855 10.1007/s12519-012-0372-2

[29] Asbury MR , Unger S, Kiss A, et al Optimizing the growth of very-low-birth-weight infants requires targeting both nutritional and nonnutritional modifiable factors specific to stage of hospitalization. Am J Clin Nutr. 2019;110(6):1384–1394. 31536118 10.1093/ajcn/nqz227 PMC6885476

[30] O’Connor DL , Gibbins S, Kiss A, et al; for the GTA DoMINO Feeding Group. Effect of supplemental donor human milk compared with preterm formula on neurodevelopment of very low-birth-weight infants at 18 months: A randomized clinical trial. JAMA 2016;316(18):1897. 27825008 10.1001/jama.2016.16144

[31] Unger S , Gibbins S, Zupancic J, O’Connor DL. DoMINO: Donor milk for improved neurodevelopmental outcomes. BMC Pediatr. 2014;14:123. 24884424 10.1186/1471-2431-14-123 PMC4032387

[32] Ng DVY , Unger S, Asbury M, et al Neonatal morbidity count is associated with a reduced likelihood of achieving recommendations for protein, lipid, and energy in very low birth weight infants: A prospective cohort study. J Parenter Enter Nutr. 2018;42(3):623–632. 10.1177/0148607117710441 28537798

[33] McGee M , Unger S, Hamilton J, et al Adiposity and fat-free mass of children born with very low birth weight do not differ in children fed supplemental donor milk compared with those fed preterm formula. J Nutr. 2020;150(2):331–339. 31599955 10.1093/jn/nxz234

[34] Ng DVY , Brennan-Donnan J, Unger S, et al How close are we to achieving energy and nutrient goals for very low birth weight infants in the first week? J Parenter Enter Nutr. 2017;41(3):500–506. 10.1177/0148607115594674 26160253

[35] Stoltz Sjöström E , Öhlund I, Ahlsson F, et al Nutrient intakes independently affect growth in extremely preterm infants: Results from a population-based study. Acta Paediatr Int J Paediatr. 2013;102(11):1067–1074. 10.1111/apa.12359 23855971

[36] Koletzko B , Poindexter B, Uauy R. Recommended nutrient intake levels for stable, fully enterally fed very low birth weight infants. World Rev Nutr Diet. 2014;110:297–299. 24751638 10.1159/000360195

[37] Wechsler D. Wechsler preschool and primary scale of intelligence. 4th ed. San Antonio, TX: The Psychological Corporation; 2012.

[38] Veraart J , Fieremans E, Jelescu IO, Knoll F, Novikov DS. Gibbs ringing in diffusion MRI. Magn Reson Med. 2016;76(1):301–314. 26257388 10.1002/mrm.25866 PMC4915073

[39] Kellner E , Dhital B, Kiselev VG, Reisert M. Gibbs-ringing artifact removal based on local subvoxel-shifts. Magn Reson Med. 2016;76(5):1574–1581. 26745823 10.1002/mrm.26054

[40] Andersson JLR , Sotiropoulos SN. An integrated approach to correction for off-resonance effects and subject movement in diffusion MR imaging. Neuroimage. 2016;125:1063–1078. 26481672 10.1016/j.neuroimage.2015.10.019 PMC4692656

[41] Tustison NJ , Avants BB, Cook PA, et al N4ITK: Improved N3 bias correction. IEEE Trans Med Imaging. 2010;29(6):1310–1320. 20378467 10.1109/TMI.2010.2046908 PMC3071855

[42] Smith SM , Jenkinson M, Johansen-Berg H, et al Tract-based spatial statistics: Voxelwise analysis of multi-subject diffusion data. Neuroimage. 2006;31(4):1487–1505. 16624579 10.1016/j.neuroimage.2006.02.024

[43] Anderson MJ , Robinson J. Permutation tests for linear models. Aust New Zeal J Stat. 2001;43(1):75–88.

[44] Smith SM , Nichols TE. Threshold-free cluster enhancement: Addressing problems of smoothing, threshold dependence and localisation in cluster inference. Neuroimage. 2009;44(1):83–98. 18501637 10.1016/j.neuroimage.2008.03.061

[45] Miller GA , Chapman JP. Misunderstanding analysis of covariance. J Abnorm Psychol. 2001;110(1):40–48. 11261398 10.1037//0021-843x.110.1.40

[46] Mori S , Wakana S, Van Zijl PCM, Nagai-Poetscher LM. MRI atlas of human white matter. Amsterdam, The Netherlands: Elsevier. 2005.

[47] Cormack BE , Harding JE, Miller SP, Bloomfield FH. The influence of early nutrition on brain growth and neurodevelopment in extremely preterm babies: A narrative review. Nutrients. 11(9):092029. 10.3390/nu11092029 PMC6770288 31480225

[48] Morgan C , McGowan P, Herwitker S, Hart AE, Turner MA. Postnatal head growth in preterm infants: A randomized controlled parenteral nutrition study. Pediatrics. 2014;133(1):e120–e128. 24379229 10.1542/peds.2013-2207

[49] Cheong JLY , Hunt RW, Anderson PJ, et al Head growth in preterm infants: Correlation with magnetic resonance imaging and neurodevelopmental outcome. Pediatrics. 2008;121(6):e1534–e1540. 18519457 10.1542/peds.2007-2671

[50] Tottman AC , Alsweiler JM, Bloomfield FH, et al Relationships between early neonatal nutrition and neurodevelopment at school age in children born very preterm. J Pediatr Gastroenterol Nutr. 2020;70(1):72–78. 31449172 10.1097/MPG.0000000000002471

[51] Blanco CL , Gong AK, Schoolfield J, et al Impact of early and high amino acid supplementation on ELBW infants at 2 years. J Pediatr Gastroenterol Nutr. 2012;54(5):601–607. 22228000 10.1097/MPG.0b013e31824887a0

[52] Blanco CL , Falck A, Green BK, Cornell JE, Gong AK. Metabolic responses to early and high protein supplementation in a randomized trial evaluating the prevention of hyperkalemia in extremely low birth weight infants. J Pediatr. 2008;153(4):535–540. 18589451 10.1016/j.jpeds.2008.04.059

[53] Ramel SE , Georgieff MK. Preterm nutrition and the brain. World Rev Nutr Diet. 2014;110:190–200. 24751630 10.1159/000358467

[54] Ferriero DM , Miller SP. Imaging selective vulnerability in the developing nervous system. J Anat. 2010;217(4):429–435. 20408904 10.1111/j.1469-7580.2010.01226.x PMC2992418

[55] Dubois J , Dehaene-Lambertz G, Kulikova S, Poupon C, Hüppi PS, Hertz-Pannier L. The early development of brain white matter: A review of imaging studies in fetuses, newborns and infants. Neuroscience. 2014;276:48–71. 24378955 10.1016/j.neuroscience.2013.12.044

[56] Tam EWY , Chau V, Barkovich AJ, et al Early postnatal docosahexaenoic acid levels and improved preterm brain development. Pediatr Res. 2016;79(5):723–730. 26761122 10.1038/pr.2016.11 PMC4853254

[57] Lechner BE , Vohr BR. Neurodevelopmental outcomes of preterm infants fed human milk: A systematic review. Clin Perinatol. 2017;44(1):69–83. 28159210 10.1016/j.clp.2016.11.004

[58] Schulzke SM , Patole SK, Simmer K. Longchain polyunsaturated fatty acid supplementation in preterm infants. Cochrane Database Syst Rev. 2011;2:CD000375. 10.1002/14651858.CD000375.pub2 14973956

[59] Anderson JW , Johnstone BM, Remley DT. Breast-feeding and cognitive development: A meta-analysis. Am J Clin Nutr. 1999;70(4):525–535. 10500022 10.1093/ajcn/70.4.525

[60] Belfort MB , Anderson PJ, Nowak VA, et al Breast milk feeding, brain development, and neurocognitive outcomes: A 7-year longitudinal study in infants born at less than 30 weeks’ gestation. J Pediatr. 2016;177:133–139.e1. 27480198 10.1016/j.jpeds.2016.06.045 PMC5037020

[61] Madore LS , Bora S, Erdei C, Jumani T, Dengos AR, Sen S. Effects of donor breastmilk feeding on growth and early neurodevelopmental outcomes in preterm infants: An observational study. Clin Ther. 2017;39(6):1210–1220. 28576299 10.1016/j.clinthera.2017.05.341

[62] Counsell SJ , Edwards AD, Chew ATM, et al Specific relations between neurodevelopmental abilities and white matter microstructure in children born preterm. Brain. 2008;131(12):3201–3208. 18952670 10.1093/brain/awn268

[63] Duerden EG , Foong J, Chau V, et al Tract-based spatial statistics in preterm-born neonates predicts cognitive and motor outcomes at 18 months. Am J Neuroradiol. 2015;36(8):1565–1571. 25929880 10.3174/ajnr.A4312 PMC7964703

[64] Demetriou A , Christou C, Spanoudis G, Platsidou M. The development of mental processing: Efficiency, working memory, and thinking. Monogr Soc Res Child Dev. 2002;67(1):1–155. 12360826

[65] Kail R. Developmental change in speed of processing during childhood and adolescence. Psychol Bull. 1991;109(3):490–501. 2062981 10.1037/0033-2909.109.3.490

[66] Rose SA , Feldman JF, Jankowski JJ. Information processing in toddlers: Continuity from infancy and persistence of preterm deficits. Intelligence. 2009;37(3):311–320. 20161244 10.1016/j.intell.2009.02.002 PMC2706531

[67] Rose SA , Feldman JF. Memory and processing speed in preterm children at eleven years: A comparison with full-terms. Child Dev. 1996;67(5):2005–2021. 9022226

[68] Anderson PJ. Neuropsychological outcomes of children born very preterm. Semin Fetal Neonatal Med. 2014;19(2):90–96. 24361279 10.1016/j.siny.2013.11.012

[69] Lee SK , Beltempo M, McMillan DD, et al Outcomes and care practices for preterm infants born at less than 33 weeks’ gestation: A quality-improvement study. CMAJ. 2020;192(4):E81–E91. 31988152 10.1503/cmaj.190940 PMC6989018

