# Supplementary material for: Early nutrition and white matter microstructure in children born very low birth weight
Source: Brain Commun. 2021 Apr 1;3(2):fcab066. doi: 10.1093/braincomms/fcab066 (PMC8100003; doi:10.1093/braincomms/fcab066)
Supplement: fcab066_Supplementary_Data [file fcab066_supplementary_data.docx]

**Supplemental Materials:**

**
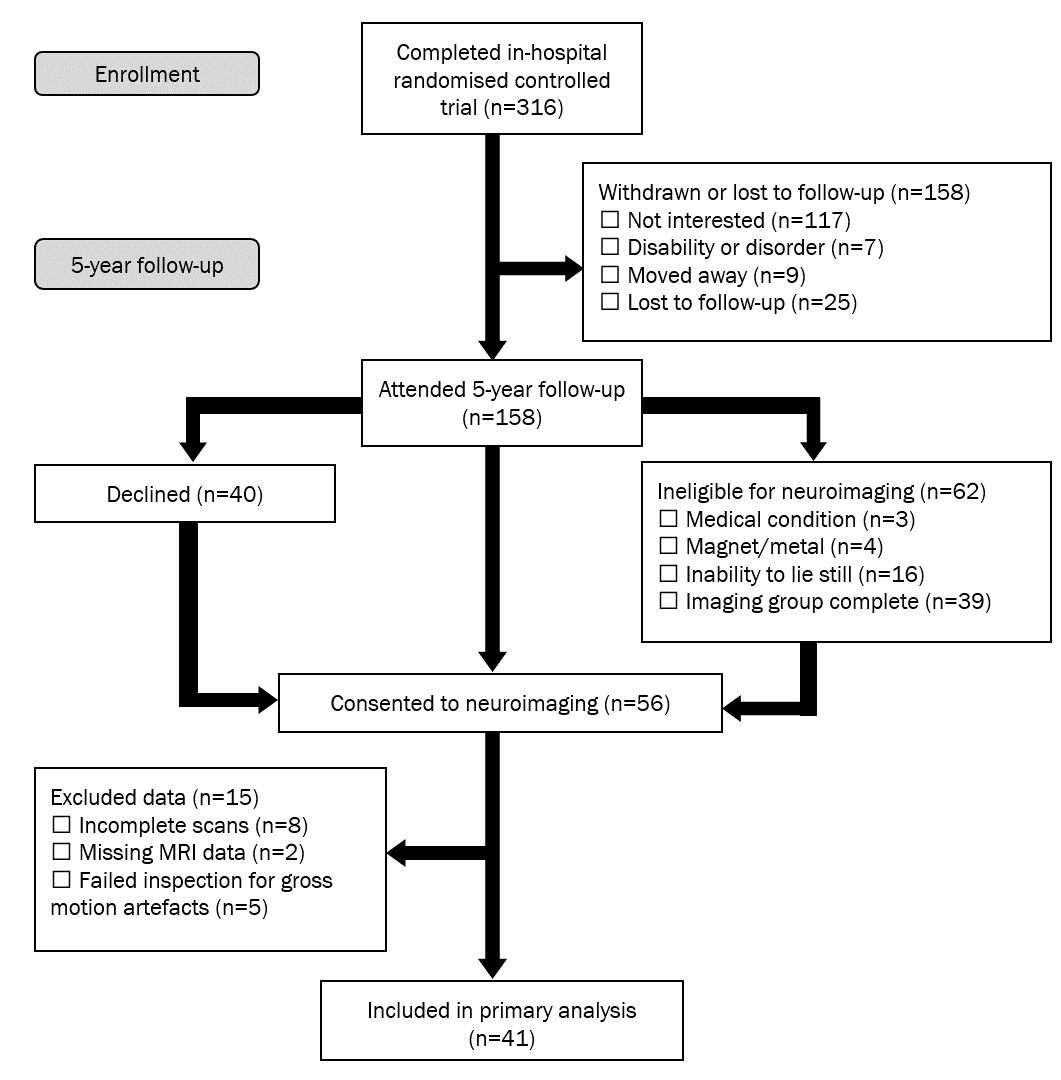
**

**Supplemental Fig. 1.** Flow of VLBW participants through the randomised clinical trial from initial enrollment to inclusion in primary analysis.


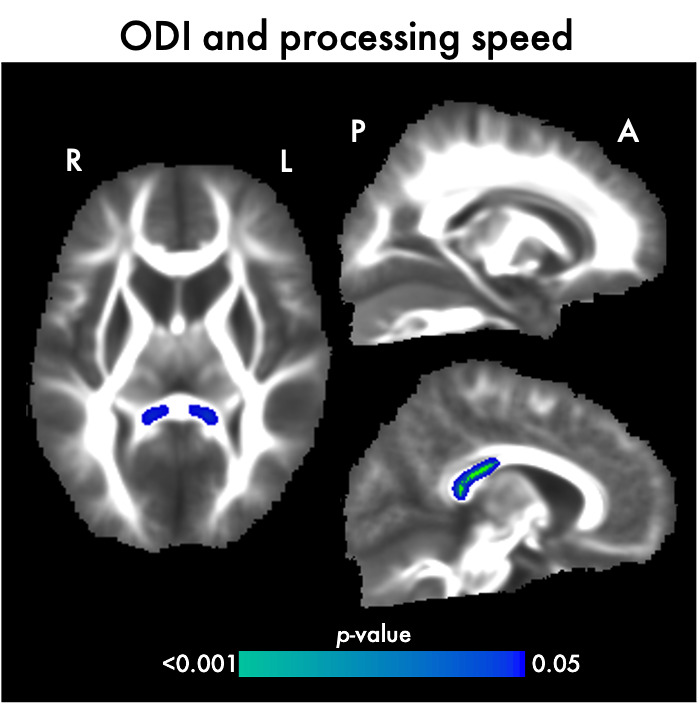


**Supplemental Fig. 2. Association between ODI and processing speed scores**. TBSS analyses demonstrated a negative association between ODI and processing speed scores, as indicated by the blue areas representing significant voxels. Significance was held at *p_corr_*<0.05. Colour bars indicate p-values.

**Supplemental Table 1**. Correlation coefficients between nutrient recommendations and measures of prematurity in VLBW children.

| VLBW group (n=40) | | | |
| --- | --- | --- | --- |
|  | Meeting protein recommendations, postnatal days 9-29 | Meeting lipid recommendations, postnatal days 9-29 | Meeting energy recommendations, postnatal days 9-29 |
| Birth weight (g) | *r=*0.43  (*p=*0.006) | *r=*0.52  (*p=*0.001) | *r=*0.47  (*p=*0.002) |
| Birth GA (weeks) | *r=*0.25  (*p=*0.120) | *r=*0.23  (*p=*0.154) | *r=*0.28  (*p=*0.081) |

**Supplemental Table 2.** TBSS analyses: Associations between DTI/NODDI metrics and cognitive outcomes in VLBW children.

| **White matter region** | **Hemi** | **+FA/**  **+PSI** | **-RD/**  **+ PSI** | **+NDI/**  **+ PSI** | **-ODI/**  **+ PSI** |
| --- | --- | --- | --- | --- | --- |
| Genu of corpus callosum | - | - | - | 49 | - |
| Body of corpus callosum | - | 1184 | 849 | 905 | 377 |
| Splenium of corpus callosum | - | 1840 | 1862 | 1234 | 530 |
| Fornix (column and body) | - | 73 | 59 | - | - |
| Corticospinal tract | R | 184 | - | 8 | - |
|  | L | 32 | 18 | 18 | - |
| Cerebral peduncle | R | 345 | 255 | 262 | - |
|  | L | 442 | 333 | 176 | - |
| Anterior limb of internal capsule | R | 8 | 6 | 36 | - |
|  | L | - | - | - | - |
| Posterior limb of internal capsule | R | 365 | 190 | 200 | - |
|  | L | 226 | 125 | 194 | - |
| Retrolenticular part of internal capsule | R | 123 | 149 | 293 | - |
|  | L | 163 | 188 | 168 | - |
| Anterior corona radiata | R | - | - | 850 | - |
|  | L | - | - | - | - |
| Superior corona radiata | R | 165 | 216 | 793 | - |
|  | L | 49 | 126 | 359 | - |
| Posterior corona radiata | R | 386 | 482 | 350 | - |
|  | L | 208 | 380 | 276 | - |
| Posterior thalamic radiation | R | 421 | 311 | 253 | - |
|  | L | 452 | 371 | 227 | - |
| Sagittal stratum | R | 39 | 8 | 55 | - |
|  | L | 1 | 77 | 39 | - |
| External capsule | R | 8 | 1 | 30 | - |
|  | L | - | - | - | - |
| Cingulum (cingulate gyrus) | R | 23 | 14 | 8 | - |
|  | L | 53 | 5 | 24 | - |
| Cingulum (hippocampus) | R | 59 | 3 | - | - |
|  | L | 7 | - | - | - |
| Superior longitudinal fasciculus | R | 173 | 365 | 488 | - |
|  | L | - | 54 | 522 | - |
| Superior fronto-occipital fasciculus | R | - | - | 2 | - |
|  | L | - | 3 | - | - |
| Uncinate fasciculus | R | - | - | - | - |
|  | L | - | - | - | - |

This table shows the number of significant voxels per region. Abbreviations: Processing speed index (PSI), Vocabulary Acquisition index (VAI), Working Memory index (WMI).
